# Analysing ethnobotanical and fishery-related importance of mangroves of the East-Godavari Delta (Andhra Pradesh, India) for conservation and management purposes

**DOI:** 10.1186/1746-4269-2-24

**Published:** 2006-05-08

**Authors:** F Dahdouh-Guebas, S Collin, D Lo Seen, P Rönnbäck, D Depommier, T Ravishankar, N Koedam

**Affiliations:** 1 Biocomplexity Research Team, c/o, Mangrove Management Group, Vrije Universiteit Brussel, Pleinlaan 2, B-1050 Brussels, Belgium; 2 Laboratory of General Botany and Nature Management, Mangrove Management Group, Vrije Universiteit Brussel, Pleinlaan 2, B-1050 Brussels, Belgium; 3 Institut Français de Pondichéry, Rue St. Louis 11, BP 33, 605001 Pondicherry, India; 4 Department of Systems Ecology, University of Stockholm, Frescati Backe, S-106 91 Stockholm, Sweden; 5 M.S. Swaminathan Research Foundation, Regional Office, 7-5A-2/1 Gopalakrishna Street, Ramaraopet, 533004 Kakinada, Andhra Pradesh, India

## Abstract

Mangrove forests, though essentially common and wide-spread, are highly threatened. Local societies along with their knowledge about the mangrove also are endangered, while they are still underrepresented as scientific research topics. With the present study we document local utilization patterns, and perception of ecosystem change. We illustrate how information generated by ethnobiological research can be used to strengthen the management of the ecosystem. This study was conducted in the Godavari mangrove forest located in the East-Godavari District of the state Andhra Pradesh in India, where mangroves have been degrading due to over-exploitation, extensive development of aquaculture, and pollution from rural and urbanized areas (Kakinada).

One hundred interviews were carried out among the fisherfolk population present in two mangrove zones in the study area, a wildlife sanctuary with strong conservation status and an adjacent zone. Results from the interviews indicated that *Avicennia marina *(Forsk.) Vierh., a dominant species in the Godavari mangroves, is used most frequently as firewood and for construction. Multiple products of the mangrove included the bark of *Ceriops decandra *(Griff.) Ding Hou to dye the fishing nets and improve their durability, the bark of *Aegiceras corniculatum *(L.) Blanco to poison and catch fish, and the leaves of *Avicennia *spp. and *Excoecaria agallocha *L. as fodder for cattle. No medicinal uses of true mangrove species were reported, but there were a few traditional uses for mangrove associates. Utilization patterns varied in the two zones that we investigated, most likely due to differences in their ecology and legal status. The findings are discussed in relation with the demographic and socio-economic traits of the fisherfolk communities of the Godavari mangroves and indicate a clear dependency of their livelihood on the mangrove forest.

Reported changes in the Godavari mangrove cover also differed in the two zones, with significantly less perceptions of a decrease in the protected area, as compared to the adjacent non-protected area. *A posteriori *comparisons between sequential satellite imagery (retrospective till 1977) and respondents that were at least 15 years back then, revealed a mangrove decrease which was however perceived to different extents depending on the area with which the fishermen were familiar. While local needs had not been incorporated in the existing policy, we created a framework on how data on ethnobotanical traditions, fishery-related activities and local people's perceptions of change can be incorporated into management strategies.

## Background

Mangrove forests fulfil a number of well-documented and essential ecological functions in tropical and subtropical regions. They generate a variety of natural resources and ecosystem services that are vital to subsistence economies and sustain local and national economies [*e.g*., [1-6]]. Mangroves provide breeding, spawning, hatching and nursery grounds for both coastal and offshore fish and shellfish stocks [3,7-13]. They also serve as a physical buffer between marine and terrestrial communities [*e.g*., [14-17]]. For local peoples, mangrove supply wood and products are harvested directly within the mangrove forest. Rapid population growth and increase utilization of mangrove habitats threatens these communities. Developing sustainable management policies that also consider the subsistence requirements of local people, is a high priority (*e.g*., [18,19]), particularly in India. Socio-economic or socio-ecological studies on mangroves are becoming more and more used [*e.g*., [20]]. However, so far, few ethnobiological surveys in mangroves have been conducted, in particularly for the general documentation of mangrove ethnobiology [*e.g*., [2,4,21]], the retrospective study of ecosystem changes (*e.g*., [22-24]), and for the investigation of management issues prior to the adoption of a particular policy [*e.g*. [25-27]. The same is true for the ethnobiological aspects of the seagrass (28) and coral reef ecosystems (29), which are often adjacent to mangroves.

Mangrove cover in India is estimated to be around 6,700 km^2 ^(30), of which 80% occurs in extensive deltaic mangrove formations along the east coast, and in the Andaman and Nicobar Islands [31]. In the State of Andhra Pradesh, a long coastline in the Districts of Krishna, Godavari East and Godavari West host natural mangrove forest along with *Casuarina equisetifolia *Forst. & Forst. plantations. The Indian mangrove flora comprises 50 species (incl. mangrove associates) and is dominated by *Avicennia *and *Rhizophora *spp., except for the Godavari wetlands, where *Rhizophora *is poorly represented [32].

The Godavari Delta, like many other deltaic systems in India, has been highly altered by human activity [32]. Since at least 1893, mangroves in this area have been subjected to heavy exploitation for fuelwood. Mangrove forests suffered heavily under various working plans until the 1978 Coringa Wildlife Sanctuary was created in the northern part of the Godavari mangrove [33,34]. The Forest Service permitted wood harvest in selected mangrove blocks. These areas were clear-cut, with the hope that the mangrove forests would regenerate naturally. Residents in nearby towns used the mangroves for agriculture, salt production and aquaculture. The Coringa Wildlife Sanctuary and other areas in the Godavari Mangrove Forest were subjected to heavy felling and feral cattle grazing, resulting in large scale depletion of the Godavari mangroves [33]. The forest is still degrading under increasing anthropogenic pressure from rural and urban areas near the city of Kakinada [35]. Causes for Godavari mangrove degradation includes conversion to aquaculture ponds, pollution, eutrophication and siltation of Kakinada Bay and its rivers, anthropogenically induced river flow change and erosion, seasonal hydrological changes, and over-exploitation by villagers [36-38]. The latter cause has lead to the current ban on wood extraction [39].

Although the current statutory provisions prohibit removal of wood, grazing of animals and establishing other activities such as shrimp farms, the Godavari mangroves are being used in an unsustainable manner [40]. Therefore, together with the Forest Department (FD) and non-governmental organisations (NGO's), the M.S. Swaminathan Research Foundation (MSSRF) initiated the Coastal Wetlands: Mangrove Conservation and Management-Project in 1997 [41]. This project empowers local people to develop subsistence policies and provides resources that serve as alternatives for mangroves (*e.g*. gas stove instead of firewood). It is within this framework that the present study fits.

To organise participatory activities community-based organisations formed the Eco-Development Committee (EDC) and the Vana Samrakshana Samithi (VSS). A subcommittee called Mangrove Restoration and Management Committee was created to ensure local's participation in the restoration project (Personal communication : Forest Department, Wildlife Conservation Rajahmundry, 2001). Handouts in Telugu (the local language) about the project were published, community meetings were held, Mangrove Clubs were formed, and illustrations on the importance of the mangroves were painted on the walls of the demonstration villages to increase local awareness [42].

The goals of the present study, carried out in a wildlife sanctuary and an adjacent non-sanctuary area, are, to acquire information on traditional uses of the mangrove ecosystem from the fishermen communities in these two areas of the Godavari mangroves, to acquire information on local perception of change, and to show how these ethnobiological data in sites with different protection status can be used to improve conservation and management of the area.

## Methods

### Description of the study site

**Figure 1:**
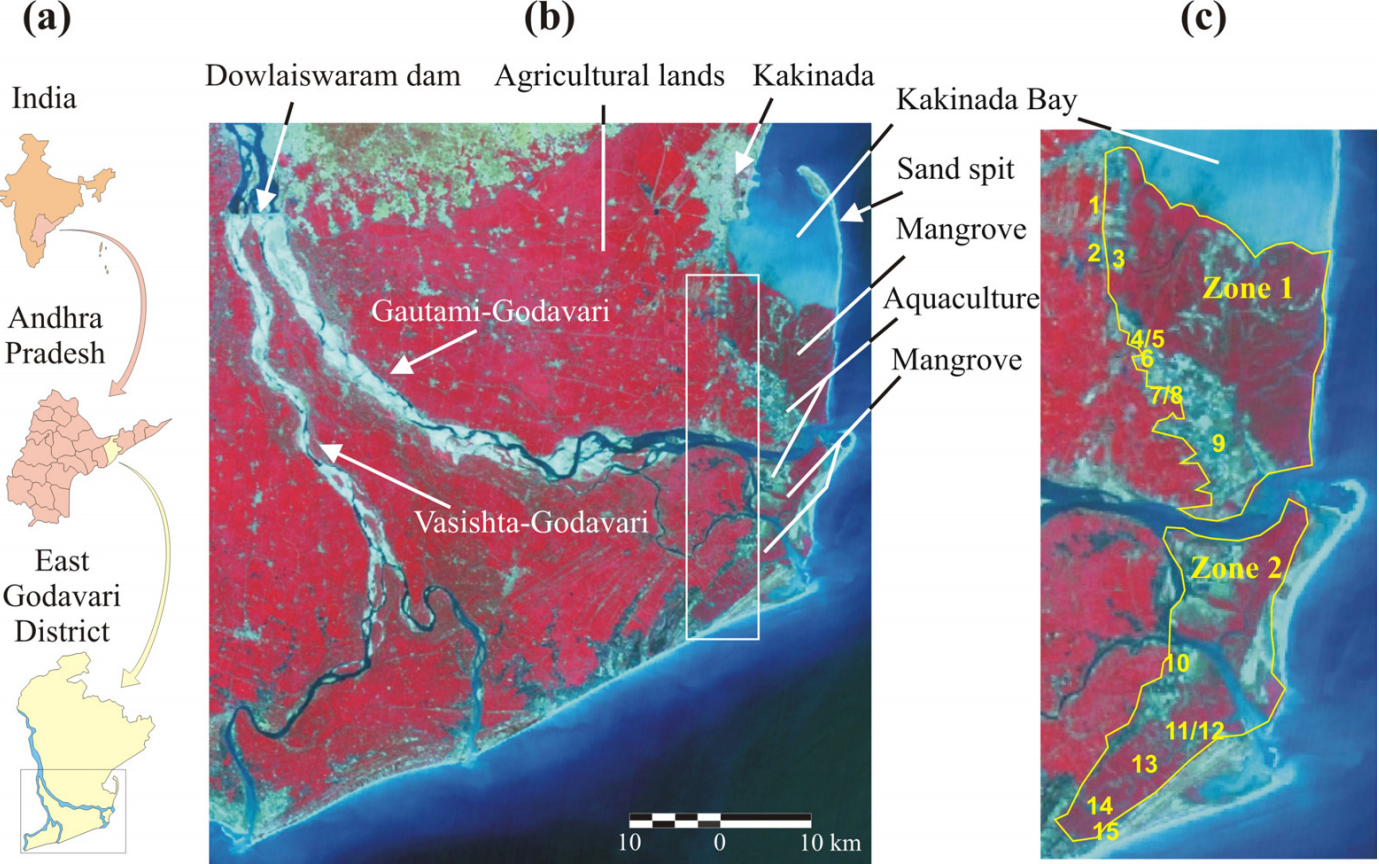
Study site. (a) Location map of India showing the state of Andhra Pradesh and the East-Godavari District (redrawn from NRSA [82]). The small black rectangle indicates the area in (b). (b) Satellite image of the Godavari Delta taken in March 1999. Adjacent to the study area, the white rectangle (*ca*. 320 km^2^) indicates the area used to extract demographic data (% fishermen) stored in the database of the South Indian Fertility Project (French Institute of Pondicherry, 2001). (c) Map of the study area investigated with the two zones and the 15 villages therein (numbered as in table 1).

The 33,263 ha Godavari mangrove wetlands are located between 16°30'-17°00'N and 82°10'-80°23'E in the East-Godavari District (Figure 1). Situated at the mouth of the 1,330 km long Godavari River (India's second longest), the Godavari mangrove forest is the second largest mangrove area on India's East Coast. It includes 15 'true mangrove species' *sensu *Jayatissa et al. [43] and Dahdouh-Guebas et al. [16]. The most important species are *Avicennia marina *(Forsk.) Vierh., *Avicennia officinalis *L., *Excoecaria agallocha *L., *Aegiceras corniculatum *(L.) Blanco, *Sonneratia apetala *Buch.-Ham., *Ceriops decandra *(Griff.) Ding Hou, *Rhizophora apiculata *Blume and *Rhizophora mucronata *Lamk. [44]. Mangrove nomenclature is following Tomlinson [45], whereas that of other species is following Mabberley [46].

**Table 1:** Criteria and their sources for the relative distinction of the Godavari mangroves into two zones, and villages studied in each zone (village numbers correspond with those in figure 1). The n-values between brackets indicate the number of questionnaires per village used in this analysis (total = 100). The asterisk refers to Appendix 1, which provides the legal text.

**CRITERIA**	**ZONE 1**	**ZONE 2**
**Separation into Reserve Forests with restricted activities (1947 Indian Forest Law part C §20)**	yes	yes
Wildlife Sanctuary* [33]	PRESENT: Coringa Wildlife Sanctuary	ABSENT
Prohibitions*	felling of trees and any type of extraction	felling of trees and collection of green wood
Restrictions on entry*	only civil servants or people living inside allowed	also people not inhabiting the sanctuary allowed
**Mangrove density**	higher	lower
**Mangrove species richness**	lower	higher
**Implementation of Forest Department regulations**	to a high degree	to a lesser degree
**Presence of Forest Department personnel**	strongly present	less present
**Accessibility of villages adjacent to the mangrove**	very accessible	less accessible
**Rehabilitation program/mangrove plantations**	present	absent
**Aquaculture**	present	present
**New aquaculture ponds**	less present	strongly present
**Villages sampled**	1. Chollangipetta (n = 7) 2. Kotthuru (n = 6) 3. Ramannapalem (n = 6) 4. Peddha Bodduvengatapalem (n = 5) 5. Chinna Bodduvengatapalem (n = 5) 6. Chinna Valasala (n = 6) 7. Peddha Valasala (n = 7) 8. Laksmipathipuram (n = 6) 9. Gadimoga (n = 7)	10. Balusutippe (n = 9) 11. Molletimoga (n = 6) 12. Kothapallem (n = 8) 13. Pora (n = 8) 14. Pandi-Pallam (n = 10) 15. Neellarevu (n = 4)

A major part of the Godavari mangroves is separated from the Bay of Bengal by Kakinada Bay. Two major shifts in the main course of the Godavari River and the formation of a sand spit have occurred since the construction of the Cotton Barrage at Dowlaiswaram in 1852 (Figure 1). Until the 1930s, the Godavari flowed northwards, opening into Kakinada Bay. Between the 1930s and the 1970s, its course gradually shifted southwards. Since the 1970s the Godavari River flows eastwards. These shifts can be explained by a combination factors including the flatness of the alluvial zone, variations in river flow, and frequent cyclonal activity in the area [47].

### Sampling design and methodology

We divided the Godavari mangrove area in 2 distinct zones based on *a priori *sample criteria (Table 1). The most important criterion was the differential legal protection status : Zone 1 comprised the Coringa Wildlife Sanctuary, whereas Zone 2 was a non-sanctuary area (Figure 1). We sampled the local population of nine villages in Zone 1 and six villages in Zone 2. The Hindu fishermen communities inhabiting these villages belong to the Agnikula Kshatriya caste. Their common language is Telugu, a Dravidian language, which is largely spoken in Andhra Pradesh [48]. Additional details regarding the socio-cultural background of the sampled communities can be found in Suryanarayana [48].

In each village, we randomly selected 4 to 10 households for interviews. A total of 55 households completed questionnaires in Zone 1 and 45 in Zone 2. We took the household as a sampling unit and we interviewed as described in Dahdouh-Guebas et al. [4: p 516]. We conducted interviews in Telugu, with the assistance of two English-Telugu bilingual translators native to the East-Godavari District. We assessed the mangrove knowledge of respondents with ethnobotanical questions, aided by a botanical photographic catalogue showing the tree physiognomies, leaves, fruits, flowers and seeds of each mangrove species. The rest of the semi-structured questionnaire contained both multiple choice and open-ended questions, which covered ethnobotanical and fishery-related issues, local perception of change in the mangroves, as well as personal socio-economic questions for each household (Appendix II). The questionnaire had not the aim to analyse gender issues or other within-household differentiation on the level of resource use. The survey was complemented with visual observations, and the collection of secondary data from both governmental organisations and NGOs. All fieldwork was carried out in October and November 2001.

There were no direct statistics available about the percentage of fisherfolk that we interviewed. According to the demographic data of 2000 obtained from the Mandal Offices of Tallarevu and Katrenikona a total of 34,625 people inhabited the villages surveyed, all of which had access to electricity, and, apart from Pora and Neellarevu, all villages contained a school. From the database of the South Indian Fertility Project at the French Institute of Pondicherry we calculated that the percentage of total active population (*i.e*. not schooling, not retired or not unemployed, although we acknowledge that it is possible that those classified as schooling, unemployed or retired would still be involved in fishing, catching or collecting in the mangrove, probably as an important coping strategy) in the villages adjacent to the survey area (Figure 1) constituted 36.4 % of the total population in that area, and that 15.7 % of this total active population were fishermen. There were no available data about active population within our study area, so we assumed that the proportion of total active population in our study area was not lower than the above figure for the adjacent villages. However, it is very likely that the study area had a higher proportion of fishermen, particularly in Zone 2. Considering a maximal proportion-of-fishermen-range between 15.7% and 100 %, and assuming that all members of the active population are married and divided into households with 2 parents, our survey then covered between 1.57 % and 10.11 % of the fishermen households in the study area, which is a demographically sound sampling basis.

### Statistical analyses

To analyse the questionnaire data statistically we used the χ^2^-test or the related G-test [49] when confronting various classes. These tests were most preferable as we were dealing with qualitative response classes. We did between-zone comparisons of means using t-tests. We did combinatory statistical analyses involving age by splitting the age classes in two equal groups and by confronting the upper with the lower age classes (see results). In the retrospective questions (past decade), we omitted answers from respondents below 25 years of age from further analysis, because younger cohorts could not realistically answer these questions (*e.g*. youngsters of 25 in 2001 were just born in 1977, see results for *a posteriori *comparisons with retrospective remotely sensed imagery).

## Results

### Demography

**Figure 2:**
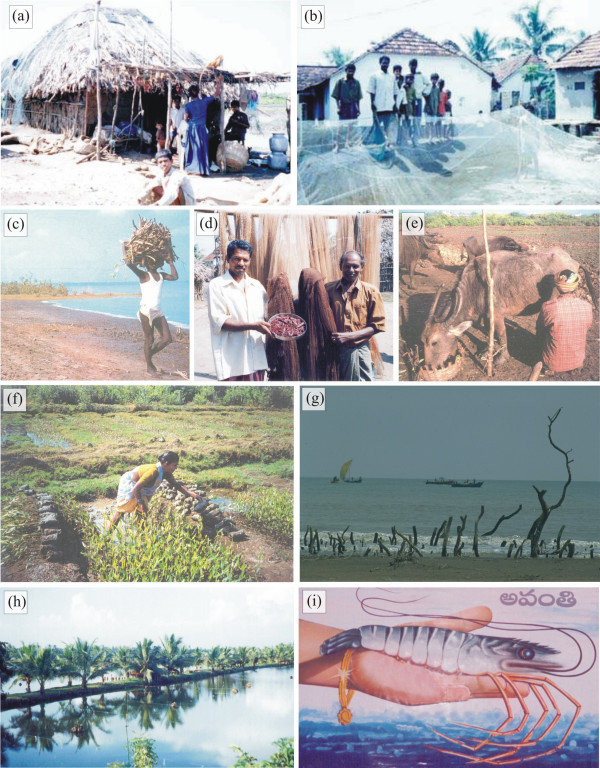
Photographs illustrating ethnobiological relationships and impacts on the mangrove. **(a) **House construction of a *kutcha *(roughly meaning 'low class'). **(b) **House construction of a *pucca *(roughly meaning 'high class'). **(c) **Traditional use of mangroves as fuelwood. **(d) **Fishermen holding a tray with pieces of *Ceriops decandra *bark used for dyeing fishing nets. They also show two freshly dyed nets and in the background previously dyed nets are hung to dry. **(e) **Herdsman milking his feral water buffalo that is consuming *Avicennia alba *twigs. **(f) **Sorting of *Avicennia *spp. seedlings in a mangrove nursery. **(g) **Although the cause of the destruction of the mangroves on the foreground is natural (cyclone 07B), the irony of this photograph is that in the background fishermen are fishing for species that are dependent on the mangrove otherwise functioning as breeding, spawning, hatching and nursery grounds. **(h) **Shrimp farm ponds established along Kakinada Road near Gadimoga (Zone 1) at the expense of mangrove forest. **(i) **Publicity in favour of shrimp farming, showing the (short-term) economic gains that may result from this activity (golden bracelet). (Photographs by Sarah Collin, Deirdre Vrancken and Nico Koedam).

The age of the fishermen interviewed ranged from 16 to 55 years old, of which 88 % was native to the villages. The main income of all the respondents originated from fishing, and ranged from less than 2,000 to more than 10,000 Indian Rupees (INR) annually (Table 2) – during the fieldwork 1 € = 43.48 INR -. A majority of the fisherfolk lived in a simple *kutcha *house (Figure 2a; Table 2) and possessed little extra items (*e.g*. farm animals, bicycle, TV). Considering this sampling homogeneity, and considering that the number of interviews per wealth class per village was low for most wealth classes we did not go into their statistical comparisons.

**Table 2:** Annual income in Indian Rupees (INR) and other assets available to the 100 fisherfolk households interviewed.

**ASSETS**	**# HOUSEHOLDS**
Annual income :	
< 2,000 INR	5
2,000 – 5,000 INR	47
5,000 – 10,000 INR	26
> 10,000 INR	9
no answer	13
Agricultural land	2
Coconut trees (*Cocos nucifera *L.)	29
Neem trees (*Azadirachta indica *A. Juss.)	1
Moringa trees (*Moringa *spp. Adans.)	4
Smallstock with goats	9
Livestock with buffaloes	3
Nava (= boat) :	
own property	40
shared property	27
rented	24
motorised	2
Bicycle	15
TV	23
Gas stove	4
Electricity	51
House type :	
*kutcha *= wood and mud hut, palm roofing (Fig. 2a)	65
semi-*kutcha *= tached hut, palm roofing	15
semi-*pucca *= tiled house	1
*pucca *= concrete house (Fig. 2b)	19

### Ethnobiology

Respondents referred to the general mangrove forest as *mada adavi*, meaning *Avicennia *forest. When inquiring about the exact meaning of 'mangrove', 56 respondents referred to the vegetation, 44 to the entire ecosystem (fauna, flora), 27 to the windbreak protecting their villages against cyclones and floods, and 8 to the direct resource (firewood, building wood, fodder).

The level of knowledge for the 13 true mangrove species encountered in this study was subdivided into 4 categories, each corresponding to a minimum number of species recognised : low (< 5 species), fair (5–7 species), good (8–10 species) and very good (> 10 species). Of all respondents, 83% had a good or very good knowledge (Table 3). When combining this level of knowledge with the age of the respondents we saw that, although there are obvious differences between the single age classes *per se*, there was no significant trend of mangrove knowledge with age (upper *versus *lower four age classes; χ^2 ^= 0.027; df = 1; p > 0.1). The level of knowledge varied across mangrove species and according to the zone the respondents lived in. Zone 1 respondents were less likely to recognize *A. marina*, *A. officinalis*, *Ceriops decandra*, *Lumnitzera racemosa *Willd., *Rhizophora mucronata *and *Xylocarpus granatum *König, and more likely to recognise *Avicennia alba *Blume, *Bruguiera gymnorrhiza *(L.) Lamk. and *Sonneratia apetala *in the same zone (6.920<χ^2^<53.875; df = 1; 2.14*10^-13 ^< p < 0.03). There were no significantly different levels of knowledge between the zones for *Aegiceras corniculatum*, *Excoecaria agallocha*, *Rhizophora apiculata *and *Sonneratia caseolaris *(L.) Engler (0.000<χ^2^< 2.296; df = 1; 0.477< p < 1.000). Another striking observation was that in Zone 2 *Avicennia alba *and the mangrove associate *Hibiscus tiliaceus *L. were unknown.

**Table 3:** Combinatory analysis of the level of knowledge of true mangrove species and the age of the respondents. Methods and results on the statistical analysis are given in the text.

**AGE CLASS**	**# RESPONDENTS (= 100%)**	**LEVEL OF KNOWLEDGE (%)**			
		**bad**	**fair**	**good**	**very good**
**16 – 20**	10	0	0	70.0	30.0
**21 – 25**	12	8.0	25.0	33.5	33.5
**26 – 30**	17	0	30.0	35.5	35.5
**31 – 35**	22	0	4.0	67.0	29.0
**36 – 40**	13	0	30.0	35.0	35.0
**41 – 45**	11	0	0	54.5	45.5
**46 – 50**	11	0	18.0	64.0	18.0
**51 – 55**	4	0	25.0	25.0	50.0

**TOTAL**	100	1.0	16.0	45.0	38.0

The respondents commonly referred to the 'use' of mangroves as a fishing area (89 %), in which they penetrate on average 10 km in Zone 2 and 15 km in Zone 1 (t = 2.25; df = 88; p < 0.05). On average, they visited mangroves for fishing 15 times per month in Zone 1 and 23 times per month in Zone 2 (t = -5.60; df = 68; p < 0.001).

**Figure 3:**
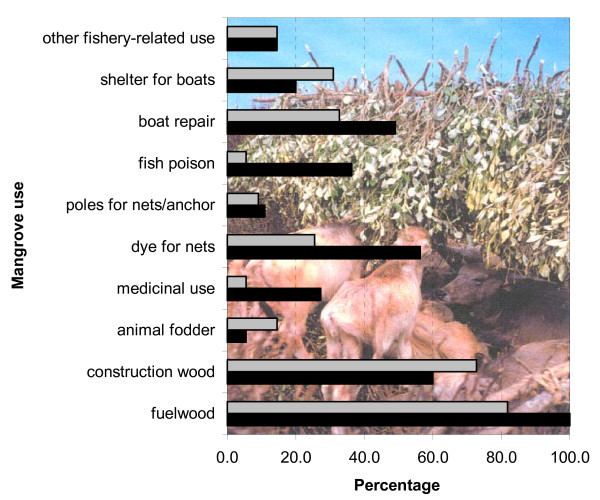
Percentage use of different mangrove use classes in Zone 1 (black) and Zone 2 (grey) amongst the 100 interviewed households (n_zone1 _= 55; n_zone2 _= 45). The background photograph shows *Avicennia *branches used as fodder for feral water buffaloes. (Photograph by Deirdre Vrancken).

Some of the uses of mangroves are illustrated in Figure 2. Among the wood and non-wood mangrove uses, a majority of the households reported the personal use of mangrove wood for fuel (Figure 2c) and construction (Figure 3). Within the construction class, respondents distinguished between poles (36% of construction use), roof beams (35 %), fences (26 %) and shelters (3 %). In addition to true mangrove species, 41% of the fishermen harvested other species for fuel, including *Borassus flabellifer *L., *Cocos nucifera *L., *Casuarina equisetifolia *and *Prosopis spicigera*, or they used sun-dried cow dung or a gas stove. However, since the true mangrove species had nearly ideal calorific values, the villagers found it difficult to use alternative resources. Likewise, 57% of the fishermen used *Borassus flabellifer *L., *Bambusa arundinacea *(Retz.) Willd. and *Casuarina equisetifolia *as alternative construction species.

There was no significant difference between the two zones for fuelwood use, but there was a significant difference in the frequency and in the distance that respondents travelled to collect it. On average, inhabitants of Zone 1 travelled 17 km 11 times per month, while those in Zone 2 travelled 27 km 5 times per month (frequency : t = -4.46; df = 55; p < 0.001, distance : t = 3.40; df = 72; p < 0.002). Zone 2 inhabitants also used significantly more mangrove as building wood (χ^2 ^= 9.065; df = 1; p < 0.01). Among the other uses (Figure 3), there were also significantly higher uses of true mangroves species or mangrove associate species for medicine (χ^2 ^= 5.792; df = 1; p < 0.02), dye for nets (χ^2 ^= 4.398; df = 1; p < 0.05) and fish poison (χ^2 ^= 10.705; df = 1; p < 0.01) in Zone 1 as compared to Zone 2. There were however no significant trends in mangrove use with age (0.004<χ^2^< 1.822; df = 1; n.s.). We also did not find differences in mangrove use between the income classes for which enough data were available (class 2,000–5,000 INR and class 5,000–10,000; see Table 2). Therefore income was not further analysed as a socio-economic factor in the light of the results presented in this paper.

Mangrove uses by species are reported in Table 4. Mangrove associates *Thespesia populnea *(L.) Solander ex. Correa and *Clerodendron inerme *(L.) Gaertn. were also used, as the most used species for boat repair (21%) and as one of the least used species for fodder (1%) respectively. Mangrove bark was employed as a dye plant (Figure 2d). Between 1 and 2 kg of *Ceriops decandra *bark was boiled in water to create a red dye to colour and increased the durability of fishing nets. This was done once or twice per month, depending of the village. The bark of *Aegiceras corniculatum *was converted into a paste and used as a fish poison. Some villagers also reported medicinal use of the mangrove associates *Caesalpinia bonduc *(L.) Roxb., *Clerodendron inerme*, *Dalbergia spinosa *Roxb., *Derris trifoliata *Lour. and *Hibiscus tiliaceus*, but no consistent data were obtained. The shopkeeper of an Ayurvedic shop in Kakinada reported that *Avicennia marina *was used as a drug against diarrhoea and dysentery, but an Ayurvedic manufacturer in Udoppa, 30 km north of Kakinada, could not confirm this. We explored local Ayurvedic literature about the topic and refer to Nadkarni [50] and Jain and Defilipps [51] for detailed information.

**Table 4:** Tree and shrub species of the Godavari mangroves and their reported multiple uses by the fishermen of the riverine villages.

		**USES**					
		**fuelwood (Fig. 2c)**	**construction wood**	**fodder (Fig. 2e)**	**boat repair**	**poles for nets/anchor**	**other uses**
**BOTANICAL NAME**	VERNACULAR NAMES IN TELUGU*						
***Aegiceras corniculatum***	*Guggilam, Dudumara*	32	2	0	0	0	fish poison
Avicennia alba	*Gundu mada, Vilava mada*	<1	0	0	0	0	no reports
***Avicennia marina***	*Tella mada*	100	60	11	9	2	no reports
***Avicennia officinalis***	*Nalla mada*	85	36	4	<1	0	no reports
***Bruguiera gymnorrhiza***	*Thuddu ponna, Uredi*	1	19	0	0	1	no reports
***Ceriops decandra***	*Gatharu, Thogara*	3	16	<1	0	0	dye/tannin for fishing nets (Fig. 2d)
***Excoecaria agallocha***	*Tilla, Tella, Chilla*	21	14	9	3	3	no reports
Lumnitzera racemosa	*Thanduga, Kadavi, Kadivi, Than*	17	25	1	13	0	no reports
***Rhizophora apiculata***	*Uppu ponna, Kaaki ponna*	2	26	0	0	5	no reports
***Rhizophora mucronata***	*Uppu ponna*	2	26	0	0	5	no reports
***Sonneratia apetala***	*Kalinga (Chinna), Kyalanki*	1	<1	1	0	0	no reports
***Sonneratia caseolaris***	*Kalinga (Peda), Kandia*	1	<1	1	0	0	no reports
***Xylocarpus granatum***	*Chenuga*	<1	<1	0	2	0	no reports

* Complemented or corrected by the nomenclature given by Pròsperi et al. [81].

In the mangrove communities, penaeid shrimps were the most important commercial catch by value (53%), followed by fish (32%), crabs (15%) and shrimp seed (1%). However, finfish catches were as important as penaeid shrimps by weight. More than 90% of the catch was sold, with no significant differences between fish, shrimps or crabs, or between zones.

## Local perception on dynamics and regulations

Seventy six percent of the fishermen of Godavari mangroves reported that the mangrove vegetation had increased over time, and they shared the opinion that this trend would continue. Seventy percent also indicated that the mangrove associate *Acanthus ilicifolius *L. had increased in vegetation cover. Among the reported reasons for the reported increase were the implementation and enforcement of Forest Department rules, a high natural regeneration, and a natural regeneration that exceeds the incidence of cutting (Fig 5). Local respondents reported that when the mangrove trees were cut, stumps would regenerate by producing new shoots. Illegal cutting of trees, mangrove conversion to aquaculture, and destructive weather phenomena (cyclones, storms) were the major reported responses for a reported decrease (Figure 5). There was no significant age trend in the proportion of people that reported an increase or decrease of the mangrove area (χ^2 ^= 0.025; df = 1; n.s.). Answers for the category of fishermen that were at least 15 years old in 1977, indicated that the answers for this category was not significantly different from the full set of data (G<0.70; df = 2; p > 0.1 n.s.). This extra test was necessary because we compared the perception of the fishermen with data based from satellite images of January 1977 (Landsat MSS), March 1993 (Landsat TM) and March 1999 (IRS LISS III) in de Solan (2001) and in VUB [39]*a posteriori*. Contrary to fishermen's perceptions, this revealed a decrease in mangrove vegetation cover. We confronted these results with the area acquainted with by the fishermen, by comparing the average distance that respondents travel for fishing (from interviews), with the remotely sensed changes that occurred within a buffer around their village with that distance as a radius. Applying GIS-technology (geographical information systems), we examined the changes in the mangrove within a 250 m margin of creek or sea separate from changes in the interior of the forest. We found that for all villages mangrove decrease largely occurred in the interior of the forest, and that colonisation (or planting; see Figure 2f) of new mangroves almost exclusively occurred along the water edge of creeks.

There was no significantly different view between people with a good to very good knowledge on mangrove species and people with a bad to fair knowledge (χ^2 ^= 1.830; df = 1; n.s.). There was, however, a clear geographical trend. The proportion of people reporting a decreasing mangrove cover was significantly larger in Zone 2 than in Zone 1 (χ^2 ^= 7.238; df = 1; 0.001<p < 0.01).

**Figure 4:**
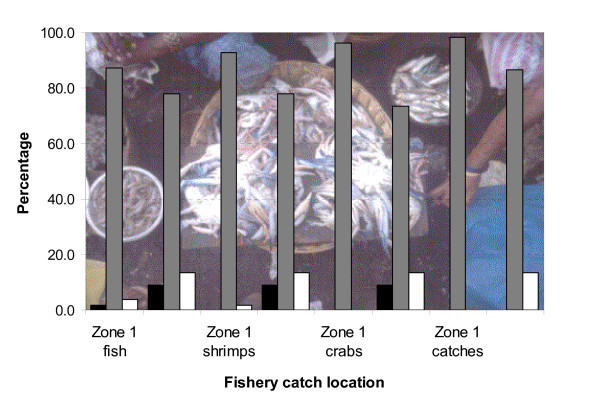
Reported perception on the changes in fish, shrimp and crab catch, and of catches in general between 1991 and 2001, in both zones (n_zone1 _= 55; n_zone2 _= 45). Black = increase; grey = decrease; white = no change. The background photograph shows crab and fish sale at a local market. (Photograph by Nico Koedam).

Fishermen unanimously reported that the catches have declined over the past 10 years (Figure 4), but the report of this decrease was significantly higher in Zone 1 as compared to Zone 2 (4.865<χ^2^< 10.277; df = 1; 0.001< p < 0.05). The causes to which the fishermen attributed this decrease cover both ecosystem-related and fishery-related issues (Figure 6).

Ninety five percent of the fishermen were aware of the Forest Department regulations. The remaining 5% entirely were from Zone II where the implementation of the rules was less pronounced, the number of Forest Department personnel was lower and the accessibility to the villages was poorer (*cf*. Table 1). Out of the 95% of fishermen that knew the rules, 97% accepted the rules because, as one respondent stated, "*a ban on cutting means an increase in mangrove cover, which is directly beneficial for the livelihood of the villagers*". But 35% of the fishermen disliked the fact that the cutting was illegal, since fuelwood was used daily for cooking and other household purposes. The high fines when caught while cutting or collecting green wood were not appreciated : 32 INR (1 € = *ca*. 44 INR in 2001), which maybe doubled or even increased by a five-fold, for one load 10 to 20 kg.

## Discussion

### Mangrove etymology

The term 'mangrove forest', '*mada adavi*' in Telugu, refers to the genus *Avicennia *(*mada*), but it is unclear whether it is the genus that adopted the name 'mangrove' because of its high abundance in this local forest (implying that in other regions, where other genera are more abundant, people would refer to the 'mangrove forest' with other names), or it is the forest in general that was named after this genus (implying that even in areas with other generic abundances people would still refer to the mangrove with the '*Avicennia*' genus). Although less logic, the latter was observed in the Teacapan-Agua Brava Lagoon in Mexico, where people regularly referred to *Laguncularia racemosa *(L.) Gaertn.f., the locally most abundant species, as '*mangle rojo*', which commonly indicates the regionally more abundant *Rhizophora mangle *L. [52]. It remains however very informative to analyse the etymology of the species or genus names, which provides insight on their popularity (knowledge by local people), ethnobotany and ecology. '*Tella mada*' (*Avicennia marina*) thus means 'White *Avicennia*', a species which in English is commonly known as the 'Grey mangrove'. '*Nalla mada*' (*Avicennia officinalis*) means 'Black *Avicennia*', a vernacular name which in English is reserved for *Avicennia germinans *(L.) Stearn.. '*Chinna*' and '*Peda*' are adjectives and mean respectively 'small' and 'large', used in the *Sonneratia *(Table 4) because the first species does not reach the heights of the second. '*Guggilam*' refers to the tree *Aegiceras corniculatum *whereas '*Guggilupu*' refers to its fruit. Also for climbing mangrove associates similar etymologies exists, such as '*Tiga*' literally meaning 'creeper' and used for *Derris trifoliata *(*Nalla tiga*).

### Socio-demographic and economic traits

With an average annual income of about 3,500 INR, fishermen are considered to be among the poorest communities in society in India [see also [40]]. Most fishermen families (65%) live in *kutcha*'s, the simplest among the four common house types (Table 2). Although this house type has been used a standard-of-living indicator, our study reveals that this may be inaccurate, since only 25% of people that earn between 2,000 and 5,000 INR annually claim to live in a *kutcha *house.

### Ethnobotany and fisheries

It is clear from the results that although the vegetation is of prime importance (*cf*. ethnobotanical uses, fisheries ground), the fishermen interpret the broader concept, function and service of the mangrove. Therefore we suggest to adopt the new term 'anthroposystem', defined as an ecosystem in which the traditional user is a subsistent ecosystem element.

Respondents do not distinguish between *Rhizophora mucronata *and *R. apiculata *(both *Ponna *or *Uppu ponna*), but they do distinguish between *Avicennia marina *(*Tella mada*) and *A.officinalis *(*Nalla mada*), with *A. alba *(*Vilava mada*) also less known (15% of respondents). Significant differences were observed between the knowledge in Zone 1 and Zone 2. This could be due to differences in abundances in the two zones. Data suggest that residents of Zone 2 visit mangrove areas more frequently than do those of Zone 1. This may lead to their greater familiarity with the species.

Although there are relatively few studies on the human uses of mangroves; publications on mangroves from Kenya [4,53,54], Tanzania [55], Vietnam [56], Mexico [24] and the Philippines [6,21], all report that construction and fuelwood are the primary uses of mangrove species. In the Godavari delta, *Avicennia *spp. and *Rhizophora *spp. are used in a mixture as poles and beams for hut building, and to construct fences and shelters (this study), but one report also highlighted the rare *Xylocarpus *to be exploited for its valuable timber [57]. In West Bengal, *Bruguiera gymnorrhiza *and *Heritiera littoralis *Dryand. have been reported as particularly valuable timber [58]. In Kenya, *Rhizophora *is favourised for house construction because of their ability to grow long and straight [4], but in the Godavari mangroves this genus is not as densely represented and rarely reaches appropriate sizes for hut building [32,44].

Although the long-standing traditional relationship with feral water buffaloes is important in the livelihood of the local people (Figure 2e) [59], and buffaloes have been observed foraging the mangrove, almost none of the fishermen (13%) admit letting their cattle graze in the forest. They claim to cut *Avicennia *spp. and *Excoecaria agallocha *leaves and bring them to the village where the cattle roam around.

Although some medicinal use of true mangrove species has been documented [2,4,60], no such medicinal use was reported in the present survey (there were however a few examples of medicinal uses for mangrove associate species). This was contrary to our expectation that was based on the legacy of Ayurvedic and plant medicine in India. We do report the use *Ceriops decandra *bark, to colour and preserve fishing nets. This traditional way of better preserving fishing nets was very relevant in the past when fishing nets were manufactured in cotton [48]. Even though most fishing nets now are made of nylon, 47% of the fishermen interviewed, continue to dye them with the red *Thogara *paste.

### Local perception on dynamics and regulations

There are scores of ethnobiological publications on resource utilisation, and sustainability [*e.g*., [62,62]]. However, the use of ethnobiological surveys in current and retrospective assessment and monitoring of natural resource status and of ecosystem change in tropical coastal ecosystems, though very promising, is novel [22,63-65]. The majority (76%) of respondents reported that the Godavari mangrove cover has increased compared with the past and they share the opinion that this trend will continue in the future. However, the Godavari mangroves have not been spared by man and have been subjected to heavy exploitation to meet local demands of fuelwood in the past [32,33]. They are still degrading due to a combination of various physical, biological and especially anthropogenical factors [36,41].

Some areas of the Godavari mangroves have been lost by conversion to shrimp farms and erosion [39,47]. During the 22-year period covered by the satellite images, a relative progression of the mangrove in the northwest into Kakinada Bay and a relative regression in the eastern parts can be noted as well [39,47]. The mangrove areas clear-felled by various working plans of the Forest Department in the past [33], were still present on the 1977 satellite image but these open areas have been regenerating successfully [47]. These observations indicate that what people perceive is not always actually being recorded with remote sensing technology. Rather than contradictory, ethnobiological data and remote sensing are complementary, and discrepancies should be interpreted in a sound framework [24,63]. The discrepancy also could be due to the fact that fishermen are acquainted with a relatively small and non-random portion of the area. Being familiar with the water edge only may be the reason why most respondents report a positive feeling about the status of the mangrove. Second, the respondents' distinctions between true mangrove species and mangrove associates may also have biased our *a posteriori *confrontation with remote sensing data. They reported for instance the dramatic expansion of the mangrove associate *Acanthus ilicifolius*, but this type of distinction of herbaceous plants is possible only with imagery with submeter spatial resolution, such as IKONOS [66]. In addition, expansion of so-called mangrove species, which in reality are mangrove associated species, may lead to misinterpretation and may mask cryptic ecological degradation in mangrove ecosystems and jeopardise functionality [16,64]. This illustrates once more that remote sensing and ethnobiological surveys are complementary and should be interpreted as such.

Fishermen reported that increased mangrove vegetation resulted from natural regeneration of cut-down stumps. However, only few species (*e.g*., *Avicennia marina, Avicennia officinalis *and *Excoecaria agallocha*) produce stump sprouts [67].

As in many areas world-wide [68-70], Andhra Pradesh has witnessed a shrimp farm industry explosion from 6,000 ha in 1990 to as much as 84,300 ha in 1999, representing more than half of the total shrimp culture area in India [20]. Often this occurs at the expense of mangroves, which function as feeding and nursery grounds [71].

Shrimp farm operations were cited as a cause for the reported decline in fish catches. A small percentage (9%) of the fishermen of the Godavari mangroves attribute aquaculture effluents as the main cause of declining harvests, but also other sources of pollution are likely to contribute [*cf*. [35]]. The devastating tropical cyclone 07B (6^th ^November 1996) with its typhoon wind speeds of 212.4 km.hr^-1 ^[72], killed 848 persons, damaged 594,000 houses, destroyed 496 ha of crops, and killed 13,507 livestock and 2,079,000 chickens and other poultry [73]. Yet only 12% of the fishermen (Figure 2g) report this to be a significant cause of declining yields. Fishermen also attribute the decline to the creation of drainage canals.

Apart from change in the mangrove area, fishermen also reported a number of fishery-related causes to declining catches. Up to 27% of the mangrove fishermen accuse their peers of overharvesting shrimp larvae, juvenile and adults (offshore trawling), leading to the decrease in catch within the mangroves. This argument is confirmed by Rönnbäck et al. [20], who report that the coastal waters surrounding the Godavari River are especially rich in penaeid shrimp resources and that trawl catches are dominated by newly emigrated juvenile and sub-adult life stages. The aquaculture-related fisheries for wild shrimp seed and broodstock support major operations in the area, but are fraught with bycatch problems [20]. Surprisingly, none of the respondents reported fish and shellfish habitat loss as a major reason behind declining catches, even though they are well aware of the role of mangroves in supporting fish and shellfish populations. Possibly the respondents were afraid of criticising aquaculture activities, and of direct conflicts with this sector.

### Use of ethnobiological data in management policy

Both scientific and societal elements should form the basis of an efficient conservation and management scheme. Such elements include biological monitoring from remote sensing [*e.g*., [19]], ecological economics [*e.g*., [5]], ethnobiological traditions and perceptions (this study), and even eco-religious approaches. With respect to the latter, Palmer and Finlay [74] paraphrases the message of the Bhagavad Gita is '*conserve ecology or perish*' – The Bhagavad Gita is the dialogue between the Hindu Lord Sri Krishna, the supreme personality of Godhead, and his intimate disciple and Prince of India Arjuna, and is considered the essence of Vedic knowledge -. These types of religious texts, which are well-known by the people, have proved to be determining elements to turn failing management policies into success [74]. Too often government policies are based only on monodisciplinary scientific studies, or, worse, just assumptions. Another shortcoming of management plans in other countries is that lack of economically acceptable alternatives for mangrove resource utilisation cause dependency [4]. Apart from scientific data many more elements can and must be used to optimalise a policy. More precisely, the policy should be at the heart of the ecologic, economic and socio-cultural reality of the communities involved. Local people are often forced to adapt to a legal conservation framework without receiving alternatives to traditional uses, or without in-depth comparative analysis of the advantages and disadvantages of the alternatives provided. Without incorporating or sufficiently studying the elements and issues of local stakeholders (food, housing, religion,...), we expect conservation and management strategies to fail.

The different views on mangrove increase or decrease from people in Zone 1 and Zone 2, can be explained by the different legal status of both zones. The implementation of the regulations set up by the Forest Department is better organised in Zone 1, which is declared as the Coringa Wildlife Sanctuary [33]. Interestingly, our results show that people inhabiting this protected area perceive more mangrove increase, whereas people inhabiting Zone 2 (which is not a sanctuary) report significantly more often a mangrove decrease. Such responses can be integrated in future management as indicators for the success of the policy with respect to mangrove conservation (Figure 7). However, the acceptance of a ban on cutting does not guarantee the social success of forest legislation, as fines are too high, and restricted access to natural resources has been reported to increase poverty in India [75]. In Kakinada, the Forest Department provides welfare measures to the villagers living around the mangrove areas to reduce their dependency on mangrove's natural resources [32]. At present, land-based alternatives for firewood, construction, fencing and fodder are provided. For the Godavari, the provision of gas stoves by the Forest Department and several NGOs is an alternative for the use of mangrove wood as fuelwood. Unfortunately wood is free, gas is not. Only 4% of the respondents of Zone I possess a gas stove. Providing such an alternative, or providing alternative wood species in artificial plantations as firewood, only works if the special characteristics of the smokeless mangrove species is taken into account.

The main cause of mangrove decrease reported by the fishermen differs across villages. In Pora (Zone II), located at the end of a long strip of mangrove area being converted to shrimp ponds, 17.5% of the respondents give this conversion as the main cause. The fishermen of Peddha Valasala (Zone I) and Neellarevu (Zone II) report the natural cause of cyclones and tropical storms. Around Peddha Valasala, there are only small mangrove patches and a continuous stretch of land is occupied by shrimp farms without any mangrove protection [76]. Neellarevu is located on an island in the mangrove forest, completely isolated from other villages. Tropical cyclone 07B (6^th ^November 1996), although devastating for the coastal villages, has been a revelation for the communities living in areas where natural mangrove forests protected residents from the cyclone fury. The answers received in Neellarevu and Peddha Valasala, where flooding damaged many of the semi-*kutcha *and semi-*pucca *houses (Figure 2a, b), claiming a high death toll (*loc. cit*.), and where previously many patches of surrounding mangrove forest were destroyed (Figure 2g), are in line with a created awareness amongst the coastal villages to preserve this unique mangrove ecosystem as a natural dyke [15,40,77].

Currently, also the very conversion from mangrove forest to shrimp farm ponds, possibly under political patronage [*cf*. [68]], and the publicity about the (short-term) economic gains involved (Figure 2i), are in strong contrast with the mangrove forest management policy. In other areas on the Indian subcontinent, mangroves are cleared to build tourist resorts which "are supposed to be located right at the beach front" (pers. obs.). This once more neglects the power of mangroves to buffer ocean surges such as from cyclones or tsunamis, and it still remains uncertain to which extent a death toll of more than a quarter million people from a single ocean surge (tsunami of 26 December 2004) will have an effect on global coastal zone management in general, and on the enforcement of local mangrove management policy in particular [16]]. Although local inhabitants foraging in the Godavari mangrove at the time of the tsunami disaster testify to have survived thanks to the mangrove (pers. comm. K. Ilangovan, French Institute of Pondicherry, January 2005), most attention from media and global organisations focused on an early warning system to announce such events without attention for early warning of mangrove degradation [*cf*. [64,66]].

Based on the present study, we made a synthesis of the elements that are used in forest management policy to find out that they did not successfully address the needs of the local communities (Figure 7). As elaborated also in the previous sections, we found for instance that quantitative information on extent of the mangrove as detected from classical remote sensing technology is primarily used to define management rules, whereas qualitative information assessed through other remote sensing tools or ethnoscientific surveys in particular, provide a better ecologic and socio-economic basis for a management policy [66,78]. We extracted the elements of our ethnobiological survey, as well as some elements from scientific literature, that point out contradictions between the policy or the alternatives provided by the government on one hand, and the effects of the laws or the evaluation of the alternatives by the local people on the other hand (Figure 7). In an Indonesian case-study Armitage [79] suggested that there is a need to formulate, propose, implement and monitor strategies that contest existing policy narratives and challenge entrenched economic interests and power relationships. It is clear that ethnobiological data, as collected and used in the present study, can be used to display contradictions and to adapt and improve the management. Although the present findings are detailed and provide a good reference on the ethnobotanical aspects of the Godavari mangroves, this type of study should be repeated in 5–10 years to assess traditional use dynamics. This would provide also useful information on the perceptions of the local fisherfolk that can be integrated in existing mangrove management plans, but also on the success of the forest management policy in the elapsed years.

## Conclusion

Tropical coastal populations, particularly in developing countries, can be highly dependent on the mangrove ecosystem for multiple purposes [2,4,80]. This statement can be elucidated by the results presented in this study, which shows that 90% of the respondents state that the Godavari mangroves are 'very important' for their livelihood. Firstly, the mangroves form a natural protection against cyclones and floods, which is realised more in villages 'facing the cyclones at the frontline'. Secondly, the mangrove ecosystem provides them with direct natural resources, such as fuel- and construction wood, fodder for the cattle and fishery-related activities. *Avicennia marina*, a dominant species in the Godavari mangroves, is most frequently used as firewood, for construction purposes and as fodder for cattle. The bark of *Ceriops decandra *is prepared traditionally to enhance the durability of the fishing nets. No medicinal use of the mangroves was reported in contrast with other areas [2,4]. Reported changes in the evolution of the Godavari mangrove cover show to be differential in two zones that differed in legal protection status, with significantly less perceptions of a decrease in the protected area, as compared to the adjacent non-protected area. Whereas, the results of our survey research indicated that elements essential to their lifestyle, have not been incorporated in the existing policy, we illustrate how data on ethnobotanical traditions, fishery-related activities and local people's perceptions of change can point out contradictions and discrepancies with the current management policy, and can therefore be used to improve the policy.

## competing interests

The author(s) declare that they have no competing interests.

## APPENDIX I: Legal text of the status of a sanctuary according to the Wildlife Act

THE WILDLIFE (PROTECTION) ACT, 1972

(No. 53 of 1972)

(9th September, 1972)

An Act to provide for the protection of [Wild animals, birds and plants]^a ^and for matters connected therewith or ancillary or incidental thereto.

^b ^[***]

CHAPTER IV

**Sanctuaries, National Park, **^1^[****] and Closed Areas

Sanctuaries

**18. Declaration of Sanctuary.- **[(l) The State Government may, by notification, declare its intention to constitute any area other than area comprised with any reserve forest or the territorial waters as a sanctuary if it considers that such area is of adequate ecological, faunal, floral, geomorphological, natural. or zoological significance, for the purpose of protecting, propagating or developing wildlife or its environment.^2^]

(2) The notification referred to in sub-section (1) shall specify, as nearly as possible, the situation and limits of such area.

*Explanation*. - For the purposes of the this section, it shall be sufficient to describe the area by roads, rivers, ridges, or other well-known or readily intelligible boundaries

**19. Collector to determine rights.- **[^3^When a notification has been issued under Sec.18,] the collector shall inquire into, and determine the existence, nature and extent of the rights of any person in or over the land comprised within the limits of the sanctuary.

**20. Bar of accrual of rights.- **After the issue of a notification under Sec."18, no right shall be acquired in, or over the land comprised within the limits of the area specified in such notification, except by succession, testamentary or intestate.

**21. Proclamation by Collector. **– When a notification has been issued under Sec.18 the Collector shall publish in the regional language in every town and village in or in the neighborhood of the area comprised therein, a progamation:

(a) specifying, as nearly as possible, the situation and the limits of the sanctuary; and

(b) requiring any person, claiming any right mentioned in Sec. 19, to prefer before the collector" within two months from the date of such proclamation, a written claim in the prescribed form specifying the nature and extent of such right, with necessary details and the amount and particulars of the compensation, if any, claimed in respect thereof.

**22. Inquiry by Collector. **– The Collector shall, after service of the prescribed notice upon the claimant, expeditiously inquire into

(a) the claim preferred before him under Cl. (b) of Sec.21, and

(b) the existence of any right mentioned in Sec.19 and not claimed under Cl.(b) of Sec.21, so far as the same may be ascertainable from the records of the State Goven-iments and the evidence of any person acquainted with the same.

**23. Powers of Collector. **– For the purpose of such inquiry, the Collector may exercise the following powers, namely

(a) the power to enter in or upon any land and to survey, demarcate, and make a map of the same or to authorise any other officer to do so;

(b) the same powers as are vested in a civil court for the trial of suits.

**24. Acquisition of rights. **– (1) In the case of a claim to a right in or over any land referred to in Sec.19, the Collector shall pass an order admitting or rejecting the same in whole or in part.

(2) If such claim is admitted in whole or in part, the Collector may either

(a) exclude such land from the limits of the proposed sanctuary, or

(b) proceed to acquire such land or rights, except where by an agreement between the owner of such land or the holder of rights and the Government the owner or holder of such rights has agreed to surrender his rights to the Government, in or over such land, and payment of such compensation, as is provided in the Land Acquisition Act, 1894 (1 of 1894)

[^4^(c) allow, in consultation with the Chief Wildlife Warden, the continuance of any right of any person in, or over any land within the limits of the sanctuary.]

**25. Acqitisition proceedings. **– (1) For the purpose of acquiring such land, or rights in or over such land,

(a) the Collector shall be deemed to be a Collector, proceeding under the Land Acquisition Act, 1894 (1 to 1894):

(b) the claimant shall be deemed to be a person interested and appearing before him in pursuance of a notice given under sec.9 of that Act.

(c) the provisions of the sections preceding Sec.9 of that Act shall be deemed to have been complied with;

(d) where the claimant does not accept the award made in his favour in the matter of compensation, he shall be deemed, within the meaning of Sec.18 of that Act, to be a person interested who has not accepted the award, and shall be entitled to proceed to claim relief, against the award under the provision of Part III of that Act;

(e) the Collector, with the consent of the claimant, or the Court, with the consent of both the parties, may award compensation in land or money or partly in land and partly in money, and

(f) in the case of the stoppage of a public way or a common pasture, the Collector may, with the previous sanction of the State Government provide for an alternative public way or common pasture, as far as may be practicable or convenient.

(2) The acquisition under this Act of any land or interest therein shall be deemed to be acquisition for a public purpose.

**26. Delegation of Collector's powers. **– The State Government may, by general or special order, direct that the powers exercisable or the functions to be performed by the Collector under Sec. 19 to 25 (both inclusive) may be'exercised and performed by such other officer as may be specified in the order.

**[**^5^**(26A) Declaration of area as Sanctuary. **-(1) When -

(a) a notification has been issued under sec.18 and the period for preferring claim has elapsed, and all claims, if any, made in relation to any land in an area intended to be declared as a sanctuary, have been disposed of by the State Government; or

(b) any area comprised within any reserve forest or any part of the territorial waters, which is considered by the State Government to be of adequate ecological, faunal, geomorphological, natural or zoological significance for the purpose of protecting, propagating or developing wildlife or its environment, is to be included in a sanctuary, the State Government shall issue a notification specifying the limits of the area which shall be comprised within the sanctuary and declare that the said area shall be sanctuary on and from such date as may be specified in the notification.

Provided that where any part of the territorial waters is to be so included, prior concurrence of the Central Government shall be obtained by the State Government.

Provided further that the limits of the area of the territorial waters to be included in the sanctuary shall be determined in consultation with the Chief Naval Hydrographer of the Central Government and after taking adequate measures to protect the occupational interests of the local fishermen.

(2) Notwithstanding anything contained in sub-section (1), the right of innocent passage of any vessel or boat through the territorial water shall not be affected by the notification issued under sub-section (1).

(3) No alteration of the boundaries of a sanctuary shall be made except on a resolution passed by the Legislation of the State.]

**27. Restriction on entry in sanctuary. - **(1) No person other than,

(a) a public servant on duty;

(b) a person who has been permitted by the Chief Wildlife Warden or the authorised officer to reside within the limits of the sanctuary;

(c) a person who has any right over immovable property within the limits of the sanctuary;

(d) a person passing through the sanctuary along a public highway, and

(e) the dependents of the person referred to in CI. (a), (b) or (c). shall enter or reside in the sanctuary, except under and in accordance with the conditions of a permit granted under section 28.

(2) Every person shall, so long as he resides in the sanctuary, be bound

(a) to prevent the commission, in the sanctuary, or an offence against this Act;

(b) where there is reason to believe that any such offence against this Act has been committed in such sanctuary, to help in discovering and arresting the offender;

(c) to report the death of any wild animal and to safeguard its remains until the Chief Wildlife Warden or the authorised officer takes charge thereof;

(d) to extinguish any fire in such sanctuary of which he has knowledge or information and to prevent from spreading by any lawful means in his power, any fire within the vicinity of such sanctuary of which he has knowledge or information; and

(e) to assist any forest officer, Chief Wildlife Warden, Wildlife Warden or police officer demanding his aid for preventing the commission of any offence against this Act or in the investigation of any such offence.

[^6^(3) No person shall, with intent to cause damage to any boundary-mark of a sanctuary or to cause any wrongful gain as defined in the Indian Penal Code (45 of 1860), alter, destroy, move, or deface such boundary-mark.]

[^7^(4) No person shall tease or molest any wild animal or litter the grounds or sanctuary.]

**28. Grant of permit. **– (1) The Chief Wildlife Warden may, on application, grant to any person a permit to enter or reside in a sanctuary for all or any of the following purposes, namely:

(a) investigation or study of wildlife and purposes ancillary or incidental thereto;

(b) photography;

(c) scientific research;

(d) tourism;

(e) transaction of lawful business with any person residing in the sanctuary.

(2) A permit to enter or reside in a sanctuary shall be issued subject to such conditions and on payment of such fee as may be prescribed.

**[**^8^**(29) Destruction, etc., in a sanctuary prohibited without a permit. **– No person shall destroy, exploit or remove any wildlife from a sanctuary or destroy or damage the habitat of any wild animal or deprive any wild animal or its habitat within such sanctuary except under and in accordance with a permit granted by the Chief Wildlife Warden and no such permit shall be granted unless the State Government being satisfied that such destruction, exploitation or removal of wildlife from the sanctuary is necessary for the improvement and better management of wildlife therein authorises the issue of such permit.

****

*Explanation: *For the purposes of this section, grazing or movement of livestock permitted under clause (d) of Sec.33 shall not be deemed to be an act prohibited under this section.]

**30. Causing fire prohibited. **– No person shall set fire to a sanctuary, or kindle any fire, or leave any fire burning, in a sanctuary, in such manner as to endanger such sanctuary.

**31 Prohibition of entry into sanctuary with weapon. **– No person shall enter a sanctuary with any weapon except with the previous permission in writing of the Chief Wildlife Warden or the authorised officer.

**32. Ban on use of injurious substances. **– No person shall use in a sanctuary, chemicals, explosives or any other substances which may cause injury to, or endanger, any wildlife in such sanctuary.

**33. Control of sanctuaries. **– The Chief Wildlife Warden shall be the authority who shall control, manage and maintain all sanctuaries and for that purpose, within the limits of any sanctuary,

(a) may construct such roads, bridges, buildings, fences or barrier gates, and carry out such other works as he may consider necessary for the purposes of such sanctuary;

(b) shall take such steps as will ensure the security of wild animals in the sanctuary and the preservation of the sanctuary and wild animals, therein;

(c) may take such measures, in the interests of wildlife, as he may consider necessary for the improvement of any habitat.

(d) may regulate, control or prohibit, in keeping with the interests of wildlife, the grazing or movement of [livestock].

(e) [omitted 1991]

**[^9^33A. Immunisation of livestock. **-(41) The Chief Wildlife Warden shall take such measures in such manner as may be prescribed, for immunisation against communicable diseases of the livestock kept in or within five kilometres of a sanctuary.

(2) No person shall take, or cause to be taken or graze, any livestock in a sanctuary without getting it immunised.]

**34. Registration of certain persons in possession of arms. **– (41) Within three months from the declaration of any area as a sanctuary, every person residing in or within ten kilometres of any such sanctuary and holding a licence granted under the Arms Act, 1959 (54 of 1959), for the possession of arms or exempted from the provisions of that Act and possessing arms, shall apply in such form, on payment of such fee, and within such time as may be prescribed, to the Chief Wildlife Warden or the authorised officer, for the registration of his name.

(2) On receipt of an application under sub-section (1), the Chief Wildlife Warden or the authorised officer shall register the name of the applicant in subject manner as may be prescribed.

[^10^3) No new licences under the Arms Act, 1959 (54 of 1959), shall be granted within a radius of ten kilometres of a sanctuary without the prior concurrence of the Chief Wildlife Warden.]

### National Parks

**35. Declaration of National Parks. **– (1) Whenever it appears to the State Government that an area, whether within a sanctuary or not, is, by reason of its ecological, faunal, floral, geomorphological, or zoological association or importance, needed to be constituted as a National Park for the purpose of protectin& propagating or developing wildlife therein or its environment, it may, by notificLtion, declare its intention to constitute such area as a National Park.

[^11^(1) Provided that where any part of the territorial waters is proposed to be included in such National Park, the provisions of Sec.26A shall, as far as may be, apply in relation to the declaration of a National Park as they apply in relation to the declaration of a sanctuary.]

(2) The notification referred to in sub-section (1) shall define the limits of the area which is intended to be declared as a National Park.

(3) Where any area is intended to be declared as a National Park, the provisions of Sec. [^12^19 to 26-A (both inclusive except clause (c) of sub-section (2) of section 24)] shall, as far as may be, apply to the investigation and determination of claims and extinguishment of rights, in relation to any land in such area as they apply to the said matters in relation to any land in a sanctuary.

(4) When the following events have occurred, namely

(a) the period for preferring claims has elapsed, and all claims, if any, made in relation to any land in an area intended to be declared as a National Park, have been disposed of by the State Government, and

(b) all rights in respect of lands proposed to be included in the National Park have become vested in the State Government the State Government shall publish a notification specifying the limits of the area which shall be comprised within the National Park and declare that the said area shall be a National Park on and from such date as may be specified in the notification.

(5) No alteration of the boundaries of a National Park shall be made except on a resolution passed by the Legislature of the State.

(6) No person shall, destroy, exploit, or remove any wildlife from a National Park or destroy or damage the habitat or any wild animal or deprive any wild animal or its habitat within such National Park except under and in accordance with a permit granted by the Chief Wildlife Warden and no such permit shall be granted unless the State Government, being satisfied that such destruction, exploitation, or removal of wildlife from the National Park is necessary for the improvement and better management of wildlife therein, authorises the issue of such permit.

(7) No grazing of any [livestock^13^] shall be permitted in a National Park and no livestock shall be allowed to enter except where such [livestock] is used as a vehicle by a person authorised to enter such National Park.

(8) The provisions of secs. 27 and 28, secs.30 to 32 (both inclusive), and CIS, (a), (b) and (c) of [Sec.33, 33A^14^] and sec.34 shall, as far as may be, apply in realtion to a National Park as they apply in relation to a sanctuary.

**36. [**^15^**Omitted 1991]**

### Closed area

**37. Declaration of closed area. **– (1) The State Government may, by notification, declare any area closed to hunting for such period as may be specified in the notification.

(2) No hunting of any wild animal shall be permitted in a closed area during the period specified in the notification referred to in sub-section(1).

### Sanctuaries or National Park declared by Central Govt

**38. Power of Central Government to declare areas as Sanctuaries or National Park, **– (1) Where the State Government leases or otherwise transfers any area under its control, not being an area within a Sanctuary, to the Central Government the Central Government may, if it is satisfied that the conditions specified in sec.18 are fulfilled in relation to the area so transferred to it, declare such area, by notification, to be a sanctuary and the provisions of [sec 18 to 35 (both inclusive)^16^], 54 and 55 shall apply in relation to such sanctuary as they apply in relation to a sanctuary declared by the State Government.

(2) The Central Government may, if it is satisfied that the conditions specified in sec.35 are fulfilled in relation to any area referred to in sub-section (1), whether or not such area has been declared, to be a sanctuary by the Central Government, or the State Government, declare such area, by notification, to be a National Park and the provisions of secs.35. 54 and 55 shall apply to such National Park as they apply in relation to a National Park declared by the State Government.

(3) In relation to a sanctuary or National Park declared by the Central Government, the powers and duties of the Chief Wildlife Warden under the section referred to in sub-section (1) and (2). shall be exercised and discharged by the Director or by such other officer as may be authorised by the Director in this behalf and references in the sections aforesaid to the State Government, shall be construed as reference to the Central Government and reference therein to the Legislation of the State shall be construed as a reference to Parliament.

---------------------------------------------------------------------

^a ^Substituted by Act 44 of 1991, sec. 2(w.e.f. 2.10.1991)

^b ^Preamble omitted by Act 44 of 1991, sec. 3.^1 ^Chapter IV "Game Reserves" omitted by Act 44 of 1991, sec. 14.

^2 ^Sec 18(l) substituted by Act 44 of 199 1, sec. 15.

^3 ^Sec. 19 "Whenever any area is declared to be a sanctuary" Substituted by Act 44 of 1991, sec. 16.

^4 ^Sec.24(2)(c) Inserted by Act 44 of 1991, sec. 17

^5 ^Sec.26A inserted by Act 44 of 1991, sec. 18.

^6 ^Sec.27(3) Inserted by Act 44 of 1991, sec. 19.

^7 ^Sec.27(4) Inserted by Act 44 of 1991, sec. 19.

^8 ^Sec.29. Hunting in sanctuary without permit phohibited. (1) Notwithstanding anything contained elsewhere in this Act, no person shall hunt any wild animal in a sanctuary or remove therefrom any wild animal, whether alive or dead, or any trophy, uncured trophy, or meat derived from such animal.

Provided that if the Chief Wildlife Warden is satisfied that it is necessary that any wild animal in a sanctuary should be hunted or removed.

(a) for the better protection of wildlife, or

(b) for any other good and sufficient reason he may, with the previous approval of the State Government, grant a permit authorising any person to hunt or remove such wild animal under the direction of an office authorised by him or cause it to by hunted or removed.

(2) A permit granted under sub-section

(1) shall specify the kind and number of wild animal that may be hunted or removed by the holder of such permit.

(3) The Chief Wildlfe Warden may, for good and sufficient reason, to be recorded in writing, cancel any permit granted under sec.28 or under this section.

Provided that no such cancellation shall he made except after giving the holder of the permit a reasonable opportunity of being heard.

(4) Any person aggrieved by the cancellation of a permit under sub-section (3) may, within 15 days from the date of such cancellation, appeal to the State Government, whose decision shall be final.

Provided that the State Government may admit any appeal preferred after the expiry of the period aforesaid if it is satisfied that the applicant had sufficient cause for not preferring the appeal in time."

Substituted by Act 44 of 1991, Sec. 20,

^9 ^Sec.33A inserted by Act 44 of 1991, sec.22.

^10 ^Sec.34(3) inserted by Act 44 of 1991, sec.22A.

^11 ^Sec.35(l) Provision added by Act 44 of 199 1, sec.23

^12 ^Sec. 35(3) " 19 to 26 (both inclusive)" between "the provisions of sections' and "shall, as far as" substituted by Act 44 of 199 1, sec.23.

^13 ^Sec.35(7) "cattle" substituted by "livestock" by Act 44 of 1991, sec.23.

^14 ^Sec.35(8) "section 33" after "clause (a), (b) and (c) of "substituted by Act 44 of 1991, sec.23.

^15 ^Sec.36 Declaration of "Game Reserve".-(1) The State Government may, by notification, declare any area closed to hunting for such period as may be specified in the notification.

(2) No hunting of any wild animal shall be permitted in such reserve except under and in accordance with a licence, issued under this section by the Chief Wildlife Warden or the authorised officer." omitted by Act 44 of 1991, sec. 24.

^16 ^Sec38. "Section 19 to 35 after "provisions of' susbstituted by Act 44 of 1991, sec.25.

This text was available from http://envfor.nic.in/legis/legis.html and from its subdirectory http://envfor.nic.in/legis/wildlife/wildlife1.html

## APPENDIX II : Ethnobiological questionnaire

Questionnaire number

Date :

Name of the village

### 1. SOCIO-DEMOGRAPHIC AND ECONOMIC TRAITS

Sex

Age

Religion

**Family status : **Bachelor/Married/Widowed

**Household size : **Male, adult (> 15 yrs)/Female, adult (> 15 yrs)/Children (< 15 yrs)/Others, allied and joint family

Since how many years are you living in this village ?

**What is your job ? **All year round ?

What are the sources of family in come (in order) ?

What is the level of annual income (Indian Rupees) of the family ?

**Which assets does the family have ? **Agricultural land (area and crops)/Trees (number and species)/Cattle (number and species)/Boat (number) (owned, shared, rented ?) (with motor ?)/Woodcart (number)/Bicycle (number)/Motorcycle (number)/TV (number)/Fridge (number)/Gas cooker/Kerosene stove/Electrical current

**Which type of house do you have ? **pucca/semi-pucca/kutcha/semi-kutcha

### 2. MAIN USES OF THE MANGROVE AS VEGETATION, AS ECOSYSTEM

**What do you understand by the term mangrove? **Vegetation, wood/The whole area, ecosystem/Other (names, concepts, uses,...)

**How many species of mangroves do you know ? **(question aided by botanical photographic catalogue)

**What are your main uses of the mangrove (in order) ? **A. Fishing (fish, prawns, crabs, shells,...)/B. Fuelwood (firewood, charcoal,...)/C. Construction and service wood (house, shelter, fence, boat)/D. Medicinal, chemical & hygienic purposes (medicine, dye, ointments,...)/E. Fodder or feed for or animals (pasture, cut-and-carry,...)/F. Others (specify)

#### 2.A. FISHING

**What do you collect from the mangrove related to fisheries (in order) ? **Crabs/Finfish/Bivalves, shells/Shrimps, prawns/Others (eggs, larvae,...)

How frequently do you go out fishing per week ?

**How far away do you travel from the village where you live to the place where you go to fish ? **(in kilometers or in time)

Are the catches of crabs, fish, shrimps for sale, for home consumption or for both ?

Has the number of best marketable fish, prawn, crab species increased or decreased over de last 10 years ? Why ?

In general, do you catch more or less than 10 years ago ? Why ?

Do you think this change is related to a change in the mangroves ? Which change ?

#### 2.B. FUELWOOD

Which species of mangrove do you use related to fuelwood (in order) and in what quantity ?

**Which are the two best species for burning and what are the criteria and characteristics making the mangrove species appropriate as fuelwood ? **High calorific value/Little or no smoke/Convenient size/Availability

If you personally collect, how frequently do you visit the mangrove to collect fuelwood ?

**How far away do you travel from the village where you live to the place where you go to collect fuelwood ? **(in kilometers or in time)

Do you collect or buy any non-mangrove fuel source ?

#### 2.C. CONSTRUCTION AND SERVICE WOOD

What species of mangrove do you use related to timber for construction purposes (in order) ? Which part of the species do you use, and for what specific purpose ?

**Which are the two best species for construction and what are the criteria and characteristics making the mangrove species appropriate as construction wood ? **High durability/Strong/Convenient in size/Abundant/Aesthetical

Do you collect or buy any non-mangrove construction items ?

#### 2.D. MEDICINAL, CHEMICAL AND HYGIENIC USE

##### What species of mangroves do you use for medicinal, chemical or hygienic purposes (in order) ? Which part of the species do you use, what is it used for, and how is it processed ?

Which local or Ayurvedic medication based on mangrove plants do you know ?

#### 3. EVOLUTION OF THE MANGROVE AREA

**How important are the mangroves for your livelihood ? **Very important/A little important/Not much important/Not at all important/I don't know

**Which changes have you noticed in the mangrove forest during your lifetime in this village (and to what can these changes be attributed ?) ? **Increase in cover/Decrease in cover/No change/I don't know

How has the accessibility of the mangrove forest changed during your lifetime in this village

**Have you noticed any changes in animal diversity in the mangrove forest during your lifetime (and to what can these changes be attributed ?) ? **The mangrove has become easier to access/The mangrove has become more difficult to access/There has been no change/I don't know

What do you think about the actual management rules implemented by the Forest Department regarding the protection and exploitation of mangroves ?

**Figure 5:**
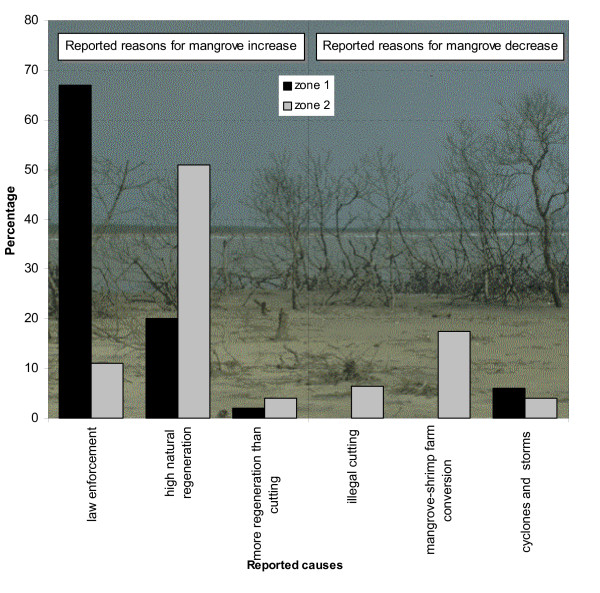
Reported causes for the reported increase and decrease in mangrove cover (n = 100). The background photograph shows the mangrove habitat for fish and shellfish destroyed by a cyclone. (Photograph by Nico Koedam).

**Figure 6:**
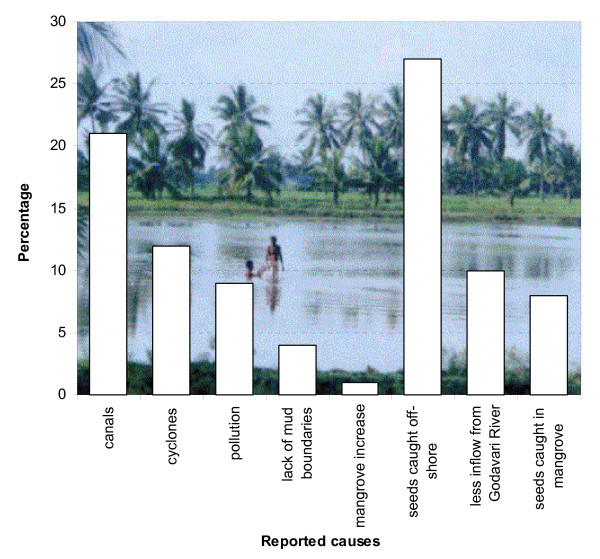
Reported causes for the reported decrease in fishery catches (n = 100). The background photograph shows the collection of shrimp seed near Gadimoga in Zone 1 (Photograph by Sarah Collin).

**Figure 7:**
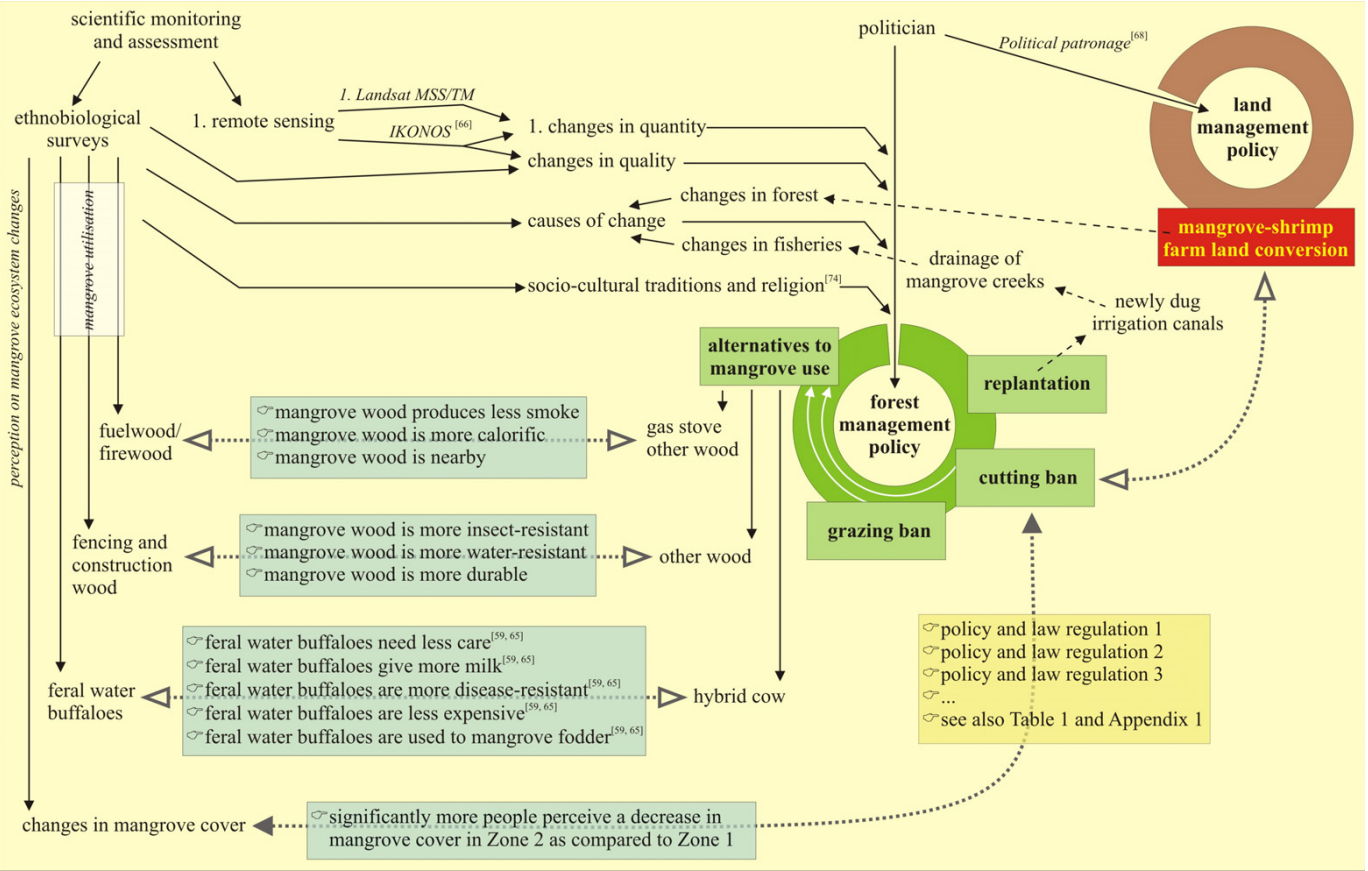
The use of ethnobiological survey data in management policy. The scheme shows forest management actions (central green circle with boxes), and what these actions are primarily based on (elements preceded by a number '1'). It also illustrates where ethnobiological elements could be used to improve the management (elements without a number). Contradictions or conformities between the management actions and the ethnobiological findings are given by the grey dotted arrows (contradiction = open arrow, conformity = closed arrow), and the boxes overlaying them provides a bulleted list with details. Unveiling such contradictions using ethnobiological surveys can help improve the policy. There is also one indication of conflict amongst policies (forest management policy *versus *land management policy), and impacts involved in the management are given as black dashed arrows. CWS = Coringa Wildlife Sanctuary. Superscripted letters refer to literature references.
